# A Novel Digital Pill System for Medication Adherence Measurement and Reporting: Usability Validation Study

**DOI:** 10.2196/30786

**Published:** 2021-11-08

**Authors:** Susan L Baumgartner, D Eric Buffkin Jr, Elise Rukavina, Jason Jones, Elizabeth Weiler, Tony C Carnes

**Affiliations:** 1 etectRx, Inc. Gainesville, FL United States; 2 Tensentric, Inc. Boulder, CO United States

**Keywords:** digital pills, digital medication, ingestible event marker, ingestible sensor, human factors, usability, validation study, medication adherence, medication nonadherence, remote patient monitoring, mobile phone

## Abstract

**Background:**

Medication nonadherence is a costly problem that is common in clinical use and clinical trials alike, with significant adverse consequences. Digital pill systems have proved to be effective and safe solutions to the challenges of nonadherence, with documented success in improving adherence and health outcomes.

**Objective:**

The aim of this human factors validation study is to evaluate a novel digital pill system, the ID-Cap System from etectRx, for usability among patient users in a simulated real-world use environment.

**Methods:**

A total of 17 patients with diverse backgrounds who regularly take oral prescription medications were recruited. After training and a period of training decay, the participants were asked to complete 12 patient-use scenarios during which errors or difficulties were logged. The participants were also interviewed about their experiences with the ID-Cap System.

**Results:**

The participants ranged in age from 27 to 74 years (mean 51 years, SD 13.8 years), and they were heterogeneous in other demographic factors as well, such as education level, handedness, and sex. In this human factors validation study, the patient users completed 97.5% (196/201) of the total use scenarios successfully; 75.1% (151/201) were completed without any failures or errors. The participants found the ID-Cap System easy to use, and they were able to accurately and proficiently record ingestion events using the device.

**Conclusions:**

The participants demonstrated the ability to safely and effectively use the ID-Cap System for its intended use. The ID-Cap System has great potential as a useful tool for encouraging medication adherence and can be easily implemented by patient users.

## Introduction

### Background

Medication nonadherence is a problem that continues to plague the health care system. Data have shown that patients do not report their own adherence accurately [1] and that health care providers are generally poor judges of their patients’ adherence [2]. In clinical practice, it is known that up to 50% of patients do not take their medications as prescribed [3], even in serious disease states or conditions where the consequences can be severe, such as diabetes, heart failure, hyperlipidemia, hypertension, and organ transplantation [4,5].

In addition, medications that have been shown to improve the quality of life, prevent tumor progression, and prolong survival are often not taken as prescribed by patients with cancer [6]. A systematic review of publications on oral anticancer medications from 2003 to 2015 showed that medication adherence rates varied widely from 46% to 100% [7]. In interviews with patients with breast cancer, de Mendoza et al [8] found that 78.9% of the patients failed to report medication discontinuation immediately and 57.9% overreported medication adherence.

Nonadherence is multifactorial. The common reasons for nonadherence are confusion (about complex drug regimens), a lack of commitment to the treatment plan, fear of adverse events, cost of drugs, forgetfulness, lack of symptoms, illness factors such as depression or psychosis, and miscommunication or lack of trust between the patient and the health care team [9-11].

Clinical trials too are often impaired by suboptimal adherence and flawed in the way they track medication adherence [12-14]. For example, in a systematic review that captured adherence data from 95 clinical trials involving 16,907 participants, there was an immediate 4% drop-off of the enrolled participants because of noninitiation of therapy. By day 100, 20% of the participants had stopped taking the medication. A further 12% displayed imperfect adherence on a daily basis [14]. Adherence errors can result in suboptimal dosing and inaccurate assessments of efficacy, safety, and tolerability, thus delaying the drug development process and potentially adding millions of dollars in additional costs [9,15].

The need for objective and reliable ways to confirm medication use has driven the development of various tracking methods, including patient self-reports, adherence-reporting mobile apps, pill counts, pharmacy prescription refill rates, electronic pill dispensers, and other solutions—but none have been optimized, and many are not reliable [16]. Digital pill systems, in contrast, have demonstrated a high rate of accuracy, with a study showing a 99.4% adherence rate across 2824 digital pill ingestions that were tracked [17].

The ID-Cap System (etectRx, Inc) is a digital pill system and ingestible event marker (Code of Federal Regulations 21 §880.6305) that enables adherence measurement through an embedded ingestible sensor. The biocompatible sensor, upon coming into contact with gastrointestinal fluid, communicates a digital signal through radio frequency after ingestion and dissolution of the pharmaceutical-grade capsule shell that encapsulates it. A reader worn by the patient detects the radio frequency signal and forwards ingestion data to the patient app and clinician dashboard. Information about the ingestion event is then wirelessly transferred to a Health Insurance Portability and Accountability Act–compliant cloud-based server [18] for secure sharing with authorized users. The sensor is naturally and safely eliminated from the body.

**Figure 1:**
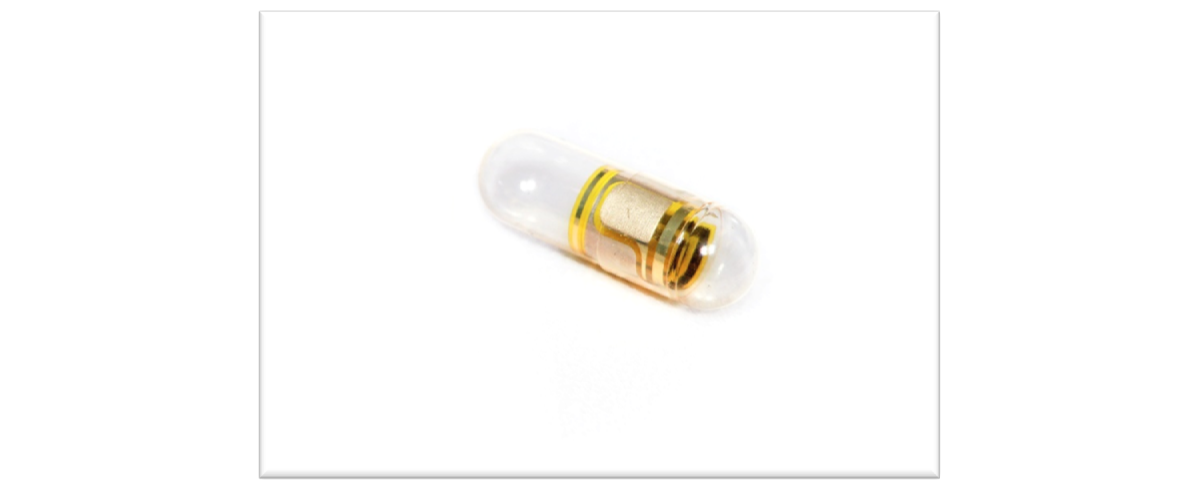
ID-Capsule: a digital pill consisting of a pharmaceutical-grade capsule shell with an embedded ingestible sensor. The ID-Capsule has been designed to encapsulate medications that are tracked using the system. The sensor communicates a digital signal shortly after ingestion and capsule dissolution. The sensor is naturally and safely eliminated through the patient’s gastrointestinal tract.

**Figure 2:**
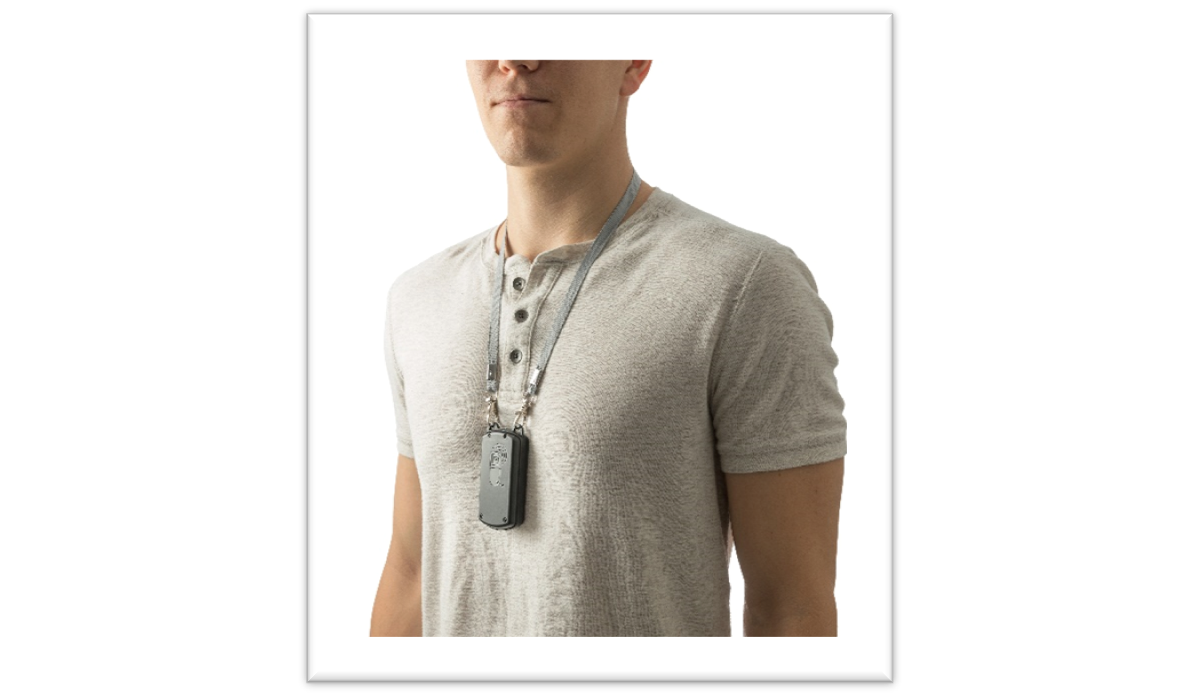
ID-Cap Reader: a wearable device that detects messages transmitted from the ingested sensor and forwards them to the ID-Cap Patient App and Clinician Dashboard.

**Figure 3:**
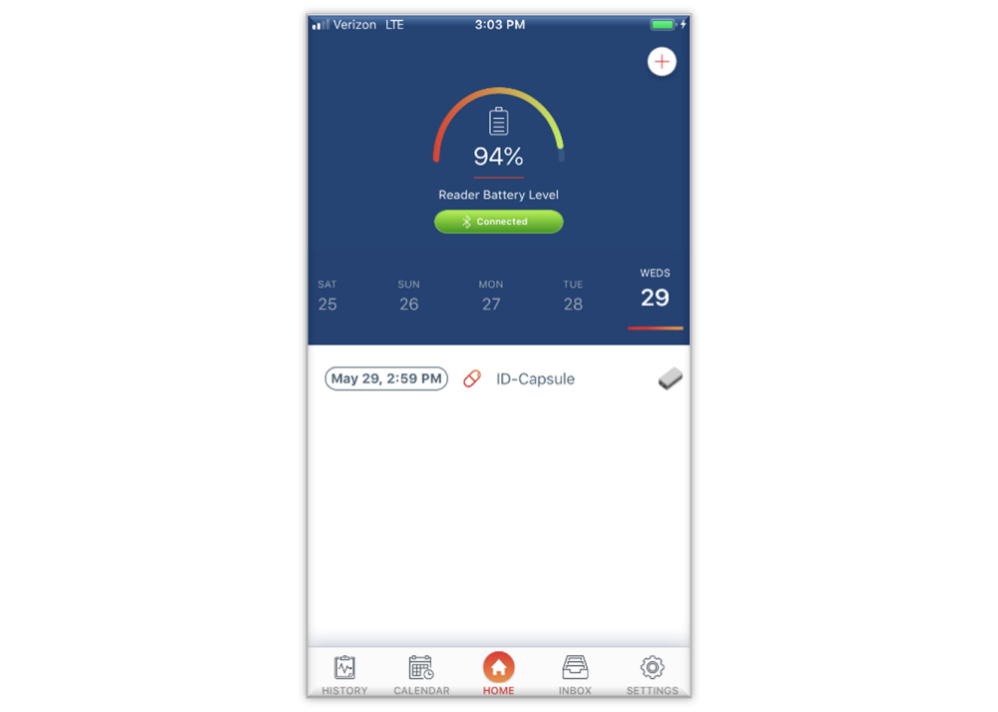
The ID-Cap Patient App allows patients to view ingestion events in real time as well as their medication use history. The app can also send patient reminders and alerts.

**Figure 4:**
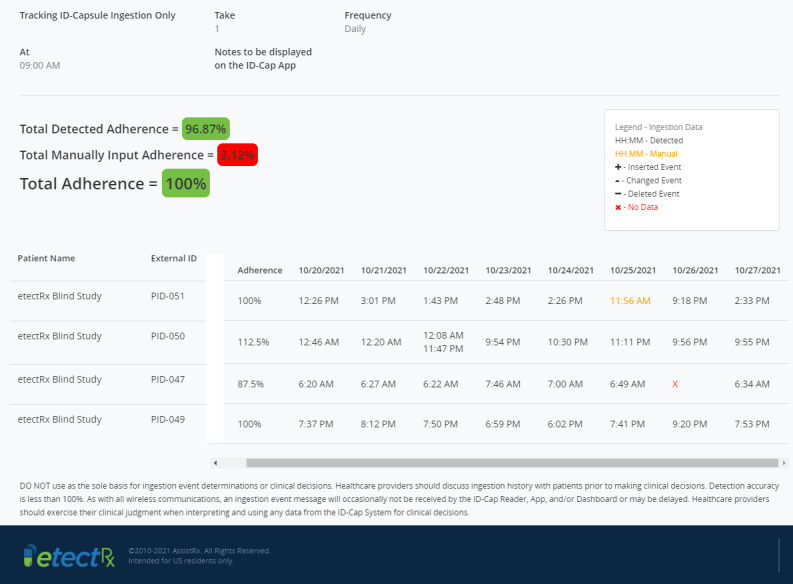
The Clinician Dashboard enables logging, tracking, and trending of patients’ ingestion events by clinicians. It provides both real-time notifications and a history of ingestion events.

**Figure 5:**
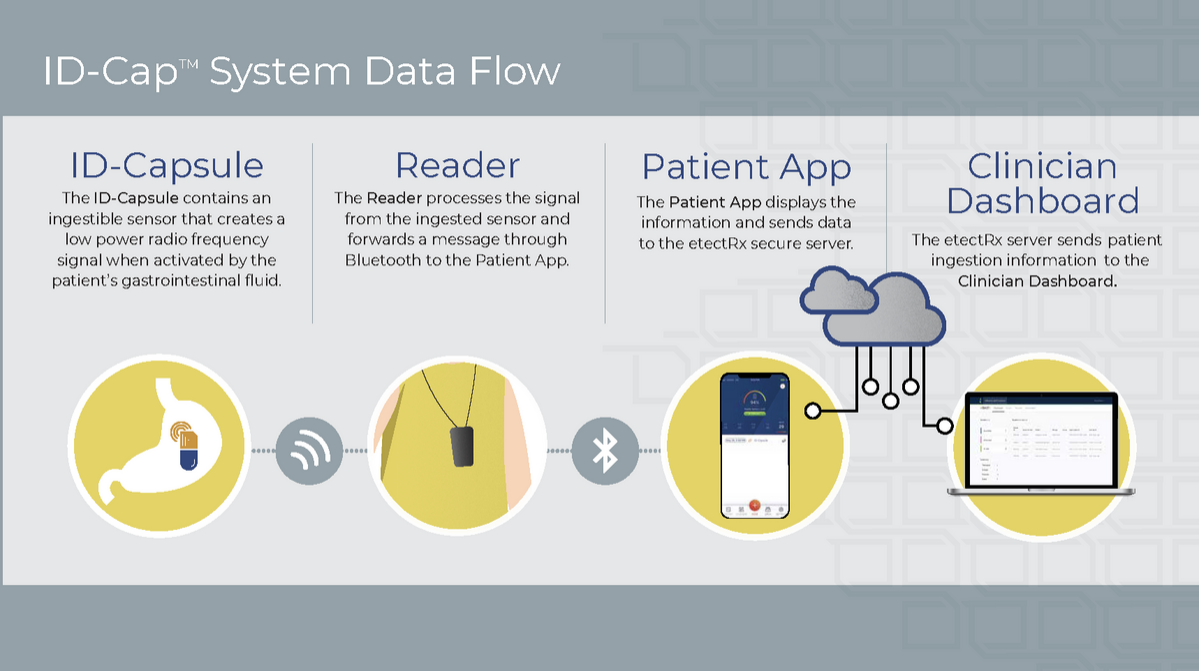
Overview of how the ID-Cap System works.

The Food and Drug Administration granted 510(k) clearance to the ID-Cap System in December 2019. The regulatory review of this medical device and its related software encompassed the results of human factors validation testing among patient users, clinician users, and system administrators, including the results of the study reported herein. The components of the ID-Cap System are shown in Figures 1-5. The patient user testing was conducted with the following system components: the ID-Capsule, the ID-Cap Reader, and the ID-Cap Patient App.

### Objective

In this paper, we describe the human factors validation study involving patient users, the intent of which is to evaluate the ID-Cap System for usability, ensuring that patient users will be able to operate the system as intended in a simulated real-world use environment. Our key questions were as follows:

1. Are patient users able to perform critical tasks effectively and safely, using an interface representative of the final device design, in conditions representing the actual conditions of use?
2. Can they do so without errors and without difficulties that could cause harm?

This validation study excluded clinical safety and effectiveness elements, which have been assessed and documented in separate evaluations and pivotal clinical trials supporting the use of the device. The researchers hypothesized a priori that the patient users would successfully demonstrate their ability to safely and effectively use the ID-Cap System for its intended use.

## Methods

### Overview

To conduct the human factors validation test of the ID-Cap System, we used assessment testing. This type of test provides users with realistic tasks to perform, using a working prototype of the device but without requiring any clinical use [19].

The training and testing took place at the office facilities of Tensentric, Inc, in Boulder, Colorado. The test was conducted in either conference rooms or dedicated research rooms set up to represent a typical home-use environment. An ID-Cap System was provided, including supplemental test equipment (eg, laptops for training videos) and product labeling.

### Participants

The research team had a recruitment target of up to 18 patient users to ensure that the goal of a minimum of 15 participants from the intended user population would be met; 15 test participants per user group represents the most stringent sample size guidance from regulatory bodies for human factors validation testing [20]. The participants were recruited by an independent third-party recruiting firm that had no knowledge of etectRx’s involvement at the time of recruitment. None of the participants were employed by, or affiliated with, etectRx, nor had they participated in a preceding formative usability or validation test of the ID-Cap System. Each participant signed a nondisclosure agreement and an informed consent form documenting their agreement for participation in the test session and video recording. Each participant received an honorarium for participation in the study, which was distributed after the completion of the test session.

Our goal was to obtain a sample of test participants who represent the intended patient users of the ID-Cap System. The recruited participants took medication by mouth on a regular basis, were able to understand and follow directions, and communicated clearly. If a caregiver assisted the participant in taking medication or in day-to-day activities, the caregiver also participated in the test. Participants with conditions that affected their ability to make health decisions or follow their physician’s instructions and those who did not use a smartphone were excluded.

This was an all-comers study that was, by design, both inclusive and diverse. Diversity within the sample size was promoted within the recruitment screener by quantified maximums or minimums on specific populations as well as instructions to recruit a variety of participants, including a mix of handedness, sexes, races, educational levels, and disease states. The demographics that were recorded after scheduling included visual acuity, age, handedness, sex, medical conditions, how the participant currently ensures that they are taking their medication, color blindness, visual impairments, and any visual corrections.

### Training

The participants received a 40-minute training session for the ID-Cap System to orient them to the basic features, functions, and nomenclature of the ingestible sensor, wearable reader, and patient app. They were shown a series of 5 short training videos containing key information and demonstrating the correct use of the device. They were then asked to demonstrate the steps for completing a successful ingestion using the reader and patient app before using the system independently during the test session. The training was equivalent to what is expected to be delivered to actual users, and the content, format, and method of delivery of training were comparable to the training that actual users would receive. The training materials and device instructions for use were designed to support a self-guided supplemental training program for the patient user.

The training videos covered the following topics:

- Overview of the ID-Cap System
- Setup of charger and reader for the first time
- Routine use of the system *without* use of the patient app
- Routine use of the system *with* use of the patient app
- App navigation and functionality

Training was conducted at least one hour before the patient’s test session to simulate a typical level of training decay. After completing the training, the participants were sent away from the test environment for at least one hour with no materials. Only after the waiting period of an hour were the participants able to begin executing the test scenarios. This 1-hour gap, which was added to be more indicative of real-world gaps that exist between training and first-time use, represents the recommended time frame when evaluating potential use-related risks related to training decay [19].

### Testing

After the training session and training decay period, the participants initiated a guided 60-minute test session. Time variation was expected in completion of the test session based on factors such as operator skill, training retention, and experience with similar devices and smartphone apps. Actual task times were documented through the video recording of each session and referenced as needed.

During the recorded test session, the participants were observed as they completed the use tasks and monitored by at least one facilitator and either a second facilitator or an observer seated in the same room as the participants. Printed instructions for use were provided to the participants and placed where they would be freely available for reference. However, the facilitator did not direct the participants to use them; this enabled insight into how patients might (or might not) refer to them during actual use.

During the test session, the participants were asked to complete 12 patient-use scenarios using the ID-Cap System, which included device use tasks and knowledge assessment tasks. They verbally read the use-scenario description that was provided on a printed note card and started the task. The participants were instructed by the facilitator to use the system as independently and naturally as possible to reflect actual use behavior. They were not informed how to complete the task or when to expect error conditions nor were they given any other information that would bias their realistic interaction with the system.

The research team observed the participants as they attempted to carry out the task scenarios and recorded any difficulties or participant comments, which were revisited with the participant during a postscenario interview. A data logger observed and logged participant behavior, user comments, and system activity. The participant was asked to simulate the ingestion of the ID-Capsule and the medication that it was intended to track during the testing. No actual ingestion events occurred during the test session.

After each use scenario, the research facilitators conducted a postscenario interview with each participant to analyze any use-related problems. The participants were prompted to provide subjective and candid assessments of any use issues experienced during the test, their probable causes, and impact.

After all task scenarios were attempted, the participants completed a postsession debriefing interview with the facilitator, where neutrally worded, open-ended questions were posed to them regarding their experience. The participants were asked to provide feedback regarding the use, safety, and usability of the ID-Cap System, as well as the clarity and effectiveness of user resources containing instructions for its use.

### Critical Tasks and Use Scenarios

This test protocol was designed to validate the critical use tasks associated with the ID-Cap System for patient users. The tasks were selected and prioritized using a risk-based analysis to cover critical tasks, safety-related tasks, frequent tasks, tasks that must be performed correctly for the device to work as intended, and key device labeling (Table 1).

The tasks that tested safety mitigations were given the highest priority; for example, tasks that have the potential to alter decisions about ID-Capsule ingestion or ingestions of medications that are taken coincident with, or co-ingested with, the ID-Capsule were determined to be the most important to evaluate. Next on the priority scale were tasks that enable proper operation of the system, followed by tasks that occur infrequently or are provided as a convenience to the user.

**Table 1:** Critical patient tasks were performed in various scenarios with the test participants, which allowed usability validation and risk assessment of the critical use tasks associated with the ID-Cap System.

Task ID	Task description
PT^a^01	Understand key device labeling for patient users
PT02	Power on reader before use
PT03	Set up charging pad and charge reader
PT08	Download and set up app on smartphone
PT09	Pair reader with smartphone
PT04	View, understand and respond to reader indicator light
PT05	Wear reader appropriately to record ingestion event
PT06	Ingest ID-Capsule (alone or co-ingested with medication)
PT07	Wear reader for sufficient time to record ingestion event
PT10	Understand and respond to ingestion confirmations from reader and app
PT11	Understand and respond to reminder notifications from app appropriately; for example, by manually recording an ingestion event that the reader did not record
PT12	View and understand ingestion history in app: App properly records a detected ingestion event Interpret reader-detected ingestions and manually-recorded ingestions

^a^PT: patient task.

### Data Analysis and Reporting

The cross-functional research team members performed a risk-based review of the human factors validation test results, including the participants’ subjective assessments of any use errors, close calls, or operational difficulties that occurred during the test. Final pass or fail determination was made based on the risk-based review of the test results and if further design modifications were required to mitigate use-related errors.

The final report summarized the test results, which included evaluation of the actual versus expected task outcomes, subjective assessments, any specific use-related problems, and recommendations for resolution.

## Results

### Demographics and Baseline Characteristics

A total of 17 participants met the recruitment criteria and were enrolled in the validation test, fulfilling the minimum target of 15 participants from the intended user population of patient users. The participants ranged in age from 27 to 74 years (mean 51 years, SD 13.8 years), and they were heterogeneous in other demographic factors as well, such as education level, handedness, and sex (Table 2). Of the 17 participants, 7 (41%) were women, and nearly one-quarter of the participants reported high school as the highest level of education attained. All participants reported taking prescription medications by mouth on a regular basis and using a smartphone. This is consistent with the expected user population for the ID-Cap System.

**Table 2:** Demographic characteristics of the patient user group (N=17).

Participant ID	Age (years)	Education	Handedness	Sex	Type of smartphone used
P01	64	High school graduate	Right	Male	iPhone 6
P02	50	Professional degree	Right	Male	iPhone 10 R
P03	44	Master’s degree	Right	Female	iPhone 7
P04^a^	41	High school graduate	Right	Male	Samsung Galaxy S10
P05	55	Professional degree	Right	Male	iPhone 6
P06	27	Bachelor’s degree	Right	Female	iPhone 6
P07	61	Bachelor’s degree	Right	Female	iPhone 8
P08	69	Professional degree	Left	Male	iPhone 7
P09	39	Bachelor’s degree	Right	Female	Samsung Galaxy S9
P10	40	Bachelor’s degree	Right	Female	iPhone 7
P11	50	High school graduate	Right	Male	Android
P12	74	Bachelor’s degree	Right	Male	LG
P13	33	Professional degree	Right	Female	iPhone XR
P14	74	Bachelor’s degree	Right	Male	iPhone 7
P15	48	Associate degree	Left	Male	Samsung Galaxy S9
P16	57	Doctorate degree	Right	Female	iPhone 8
P17	49	Some college—no degree	Left	Male	Samsung S7 Edge

^a^P04 was a wheelchair-bound quadriplegic person who was accompanied by a caregiver for the test session.

### Test Session Results

In this validation study, the participants successfully completed 97.5% (196/201) of the total patient use scenarios with the ID-Cap System. Of the 12 use scenarios, 9 (75%) were successfully completed without any failures or use errors among the 17 test participants.

The task scenarios included first-time use tasks and repeat-use tasks, as well as tasks that were only completed on a single occasion, as appropriate, to represent actual use of the system. Of note, we found that when use errors did occur, they occurred only in the first instance and were not repeated. The use errors, close calls, and patterns of use difficulties identified in the testing are reported in Table 3.

The results of the human factors validation test were reviewed by a cross-functional team that conducted a risk-based review of each use-related finding. No new use-related risks were identified in the validation test. It was determined that no modifications of the device or software were required to improve safety or usability for the intended patient users, uses, and use environments of the ID-Cap System when operated as indicated and in a manner consistent with its labeling.

**Table 3:** Use errors, close calls, and patterns of use difficulty encountered within patient scenarios and patient knowledge tasks. Successful completion rate is the percentage of participants who successfully completed the task among those who attempted it (N=17).

Scenario	Title	Successful completion rate, % (number of participants who successfully completed the task/number of participants who attempted the task)	Summary of use errors, close calls, or use difficulties
PS^a^01	Set up & confirm reader is ready for use	100 (17/17)	A few participants had difficulty turning on the reader, but all were ultimately able to do so after 1-2 minutes and without assistance from the test facilitator
PS02	Record ingestion event using the ID-Cap reader: ID-Capsule alone	88 (15/17)	Several participants took the ID-Capsule before putting the reader on. In each instance, the participants put the reader on <1 minute after simulating ingestion of the ID-Capsule. Because of the approximately 30-minute detection window, these instances would not have resulted in a missed ingestion event. In subsequent scenarios, all participants remembered to wear their reader before taking the ID-Capsule A participant showed initial difficulty in recognizing the white indicator light; in subsequent scenarios, the participant was able to recognize the white light without difficulty Another participant had difficulty recognizing the white indicator light and prematurely removed the reader during an ingestion event. This participant showed no later difficulties related to this task for the remainder of the test session Several participants had difficulty initially interpreting a blinking versus steady reader indicator light but had no further difficulty in the test session A participant wore the reader incorrectly with the gold side of the reader facing away from the body based on instruction that they thought they had received from the training videos. After referencing the quick start guide, the participant self-corrected. The training videos were reviewed, and there was only mention that the reader should be worn with the gold side facing the body
PS03	Record ingestion event using the ID-Cap reader: Co-ingested ID-Capsule with medication	100 (17/17)	Some participants placed the reader on the charging pad in the wrong orientation but self-corrected and used the correct orientation for the remainder of the test session
PS04	App & reader setup	100 (17/17)	Two participants initially had difficulty pairing the reader to the ID-Cap App because they had not turned on the reader but self-corrected after consulting the quick start guide. They were able to complete the scenario successfully A participant had difficulty understanding the iOS Bluetooth pairing request message displayed on their iPhone but was eventually able to pair the reader to the ID-Cap App successfully
PS05	Record ingestion event using the ID-Cap Reader & App: ID-Capsule alone	100 (17/17)	A participant showed difficulty in understanding the purpose and appropriateness of recording a manual ingestion event, but there is no associated safety risk with this action
PS06	Charge reader	100 (17/17)	A participant had difficulty distinguishing the blinking orange reader indicator light because of possible poor vision and possible expectancy bias because they stated that they had expected the video to show a green blinking light. The participant correctly answered the appropriate action to take if the reader light is blinking orange
PK^b^01	Interpreting key indicator light	82 (14/17)	Four participants incorrectly stated that they would place the reader on the charging pad when the reader indicator light was red. After being directed to the quick start guide, each participant was able to understand the meaning of the red light and stated that they would leave the reader off the charging pad
PS07	Record ingestion event using the ID-Cap Reader & App: Co-ingested ID-Capsule with medication	100 (17/17)	The same participant as in PS05 again showed difficulty in understanding the purpose and appropriateness of recording a manual ingestion event, but there is no associated safety risk with this action
PS08	View and interpret ingestion history in app	100 (17/17)	Two participants had difficulty interpreting the meaning of the icon used to represent a manually recorded ingestion event. After referencing the user guide, both participants were able to find, and understand the meaning of, the icon
PK02	Respond to ID-Cap App reminders – ID-Capsule alone	94 (16/17)	A participant stated that if they could not remember whether they had taken a once-daily prescribed ID-Capsule, they would take an additional ID-Capsule because they were confident about manually entering the information if the reader did not record the event and because there was no possibility that they would forget. In the following scenario, where the ID-Capsule was taken with a medication, they indicated that they would NOT take a second one and instead contact their physician
PK03	Respond to ID-Cap App reminders – Co-ingested ID-Capsule with medication	100 (16/16)^c^	None
PK04	Understand key labeling related to the system	100 (15/15)^c^	None

^a^PS: patient scenario.

^b^PK: patient knowledge.

^c^Time constraints prevented 2 participants from completing use scenarios PK03 or PK04.

### Participant Feedback

The participants grasped the potential value of the system in helping them track and report medication adherence and could see the benefits to people who take medications regularly (including themselves). Many commented on the simplicity and ease of use of the system and liked the training and user resources that were provided, especially the training videos and quick start guides.

The participants, in general, conceptually understood that they should always take their medication as prescribed, supporting the intended role of the system as an adjunct tracking system that does not replace or change physician instructions. Of the 16 participants who answered the postsession interview questions (1 participant did not complete the postsession interview), 16 (100%) stated that they felt that they could use the system effectively and safely to record and track ingestion events and 16 (100%) reported that they believed that the system is safe to use *as is* (Textbox 1).

**Selected patient verbatim quotes reflective of overall participant feedback.**

**Relevant quotes from patient users**

- “I think it’s a really great idea for people who have issues with remembering medications and for MDs tracking how they’re doing with taking those medications.” [P03]
- “Pretty slick! Pretty minimalist, which is good for the target audience. Very easy. One button [on reader] is good. Easy to use, easy to set up. Not a lot to do. Seems very user friendly.” [P09]
- “It’s a tool. For people that have to take a lot of meds, it is a good tool. I can see the value if it communicates to a provider.” [P15]
- “It’s simple to use. If everything’s working correctly, it meets its intended purpose. The videos and instructions are very good. I had no problems trying to figure it out and follow the process.” [P05]
- “Having test driven it, I have confidence in it. That equates [to] safe use.” [P05]

## Discussion

### Principal Findings

The patient’s voice has become an important one in health care. Patients are increasingly involved in decisions about their health and medical treatment, and they have become sophisticated health care technology users who understand the value of digital platforms and are eager to use them.

Certainly, the value of the ID-Cap System and other remotely deployed digital health solutions depends on the willingness of patients to engage with them and the ability of patient users to effectively, safely, and conveniently incorporate them into everyday life with minimal training and oversight. The US Food and Drug Administration requires human factors validation testing of digital pill systems and many other medical devices to ensure safety and effectiveness for a device’s users, uses, and intended use environments [20].

The results of this human factors validation study show that a representative group of patient users successfully completed the critical and safety-related use tasks necessary for optimal use of the system independently, after receiving training that was followed by a period of training decay. Although the participants were representative of a diverse group of potential patient users who regularly take oral prescription medications, the sample size in this study was limited. The participants ranged in age from 27 to 74 years (mean 51 years, SD 13.8 years), and they were heterogeneous in other demographic factors as well, such as education level (3/17, 18% reported high school graduate as highest education level), handedness (3/17, 18% were left-handed), and sex (7/17, 41% were women). This study was neither designed nor powered to evaluate differences in usability based on demographic factors, medical history, or medication use. It is important to note that nearly every screened participant, regardless of age or education level, agreed to participate in this study after receiving information about the digital pill system and successfully completed the use tasks, showing that patient users of all types adapted well to a novel digital pill system. Use errors, when they did occur, occurred only in the first instance for tasks that are repeated with use, indicating that the participants learned to use the ID-Cap System rapidly—a positive prognosticator for real-world use. Most patients expressed satisfaction with the ID-Cap System and responded favorably to questions about the ease of use and the perceived value of the system.

Prior iterations of digital pill systems used a patch-based reader that adhered to the patient’s skin. Clinical evaluations of the patch-based form factor indicated significant limitations from the patient user’s perspective with respect to tolerability and usability [17,21]. The ID-Cap System that was tested uses a reader on a lanyard. The patient users found this reader to be easy to use and acceptable in its current form. A wrist-worn reader that may be worn like a watch or may be attached or integrated into the user’s existing watch or smartwatch is currently being evaluated. Patients are not only adjusting to new therapeutic regimens but also working to develop new medication-taking behaviors and to adopt support tools and programs that will help them to be successful. Readers that are unobtrusive and can be easily incorporated into daily life will be most readily accepted [18].

The limitations of our study include the fact that this was a simulated use of the ID-Cap System and did not include the actual taking of medication for adherence tracking. The participants reported taking prescription medications by mouth on a regular basis. However, they were not asked to use the digital pill system in this study to actually track and record their own medication use in the same way that patient users would use it in the real world.

In most clinical applications, patient users would use the system chronically over extended periods of time. This validation test was limited to only a few simulated ingestion events and did not evaluate use over time. Certainly, there may be specific patient populations, medical conditions, or treatment-related effects that would affect the usability of the ID-Cap System. This study evaluated the general operation of the device across a diverse group of patient users with different health conditions and varied medication history. Use-related risks should be assessed when the device is applied to specific clinical situations and patient populations to ensure continued safe and effective use from a human factors perspective. Digital pill systems may be incorrectly used or misused by patients in clinical trials or in clinical practice; however, these aspects of use and failure modes were not specifically explored in this study. Risk analyses have been conducted by the device manufacturer to examine and mitigate risks to patients and device performance associated with device use and misuse.

The availability of a call center or additional supporting resources during the use of the device may assist patients, as would engagement with their health care provider. These resources were not evaluated in this test protocol. Patient support programs and data-driven interventions offered by the care team members, research personnel, or device manufacturer may be beneficial for the use of the ID-Cap System in clinical practice and clinical research. Continued efforts to educate clinicians and patients alike regarding the value of the information provided by the system and proper use of the system will further enhance its adoption. In addition, integration of the ID-Cap System into existing care models, electronic health records, and clinical data management systems is being explored.

There is great potential for digital pill systems such as the ID-Cap System to contribute to the efficiency and ultimate success of clinical trial programs. For example, digital pills may be used to assess the likelihood of adherent behavior among prospective trial participants. Once a trial has started, digital pills can identify patient nonadherence and changes in patterns of use early, enabling rapid intervention and course correction. Dose-finding studies for self-administered oral medications are another ideal application for digital pills because their results and outcomes are dependent on (1) human behavior as it relates to medication taking and (2) the quality of adherence measurement to optimize drug exposure and dosing decisions. The robustness of the adherence data collected with digital pills provides an added level of reassurance that the efficacy and safety results of pivotal drug development trials eventually reported are accurate. The inconsistency of medication adherence reporting within clinical trials has led to the creation of guidelines for researchers and trial sponsors on the inclusion and implementation of adherence measures in study protocols [12-14,22].

### Conclusions

The extent of medication adherence, both in clinical use and in clinical trials, is a controllable factor important for therapeutic success and drug development. The pursuit of a solution to the widespread problem of medication nonadherence has led to digital pill systems, which have shown strong performance and a high rate of accuracy. Currently, >15 years of experience and safety data support the use of digital pills with >140,000 ingestions recorded in >1000 patients who have used these devices safely and effectively [23]. In this human factors validation study, the patient users demonstrated the ability to rapidly learn how to use the ID-Cap System and to safely and effectively use the system as intended. The patient users concluded that the device was easy to use and had the potential to be a useful tool for helping to manage their medications. As health care continues to evolve toward remote care delivery and digital health solutions become ubiquitous, systems such as the ID-Cap System that are easy to use, accepted by patients, and valuable in achieving health outcomes will be indispensable.

## References

[1] Agot K, Taylor D, Corneli AL, Wang M, Ambia J, Kashuba AD, Parker C, Lemons A, Malahleha M, Lombaard J, Van Damme L (2015). Accuracy of self-report and pill-count measures of adherence in the fem-prep clinical trial: implications for future hiv-prevention trials. AIDS Behav.

[2] Kekäle M, Talvensaari Ki, Koskenvesa P, Porkka K, Airaksinen M (2014). Chronic myeloid leukemia patients' adherence to peroral tyrosine kinase inhibitors compared with adherence as estimated by their physicians. Patient Prefer Adherence.

[3] Osterberg L, Blaschke T (2005). Adherence to medication. N Engl J Med.

[4] Lloyd JT, Maresh S, Powers CA, Shrank WH, Alley DE (2019). How much does medication nonadherence cost the Medicare fee-for-service program?. Med Care.

[5] Pinsky BW, Takemoto SK, Lentine KL, Burroughs TE, Schnitzler MA, Salvalaggio PR (2009). Transplant outcomes and economic costs associated with patient noncompliance to immunosuppression. Am J Transplant.

[6] Makubate B, Donnan PT, Dewar JA, Thompson AM, McCowan C (2013). Cohort study of adherence to adjuvant endocrine therapy, breast cancer recurrence and mortality. Br J Cancer.

[7] Greer JA, Amoyal N, Nisotel L, Fishbein JN, MacDonald J, Stagl J, Lennes I, Temel JS, Safren SA, Pirl WF (2016). A systematic review of adherence to oral antineoplastic therapies. Oncologist.

[8] de Mendoza AH, Cabling ML, Dilawari A, Turner JW, Fernández N, Henderson A, Zhu Q, Gómez S, Sheppard VB (2019). Providers' perspectives on adherence to hormonal therapy in breast cancer survivors. Is there a role for the digital health feedback system?. Health Technol (Berl).

[9] Shiovitz TM, Bain EE, McCann DJ, Skolnick P, Laughren T, Hanina A, Burch D (2016). Mitigating the effects of nonadherence in clinical trials. J Clin Pharmacol.

[10] (2020). 8 reasons patients don’t take their medications. American Medical Association.

[11] Polinski JM, Kesselheim AS, Frolkis JP, Wescott P, Allen-Coleman C, Fischer MA (2014). A matter of trust: patient barriers to primary medication adherence. Health Educ Res.

[12] Vrijens B, Urquhart J (2014). Methods for measuring, enhancing, and accounting for medication adherence in clinical trials. Clin Pharmacol Ther.

[13] Murali KM, Mullan J, Chen JH, Roodenrys S, Lonergan M (2017). Medication adherence in randomized controlled trials evaluating cardiovascular or mortality outcomes in dialysis patients: a systematic review. BMC Nephrol.

[14] Blaschke TF, Osterberg L, Vrijens B, Urquhart J (2012). Adherence to medications: insights arising from studies on the unreliable link between prescribed and actual drug dosing histories. Annu Rev Pharmacol Toxicol.

[15] Eliasson L, Clifford S, Mulick A, Jackson C, Vrijens B (2020). How the EMERGE guideline on medication adherence can improve the quality of clinical trials. Br J Clin Pharmacol.

[16] Aldeer M, Martin R (2017). Medication adherence monitoring using modern technology. Proceedings of the IEEE 8th Annual Ubiquitous Computing, ElectronicsMobile Communication Conference (UEMCON).

[17] Eisenberger U, Wüthrich RP, Bock A, Ambühl P, Steiger J, Intondi A, Kuranoff S, Maier T, Green D, DiCarlo L, Feutren G, De GS (2013). Medication adherence assessment: high accuracy of the new ingestible sensor system in kidney transplants. Transplantation.

[18] Chai P, Castillo-Mancilla J, Buffkin E, Darling C, Rosen R, Horvath K, Boudreaux ED, Robbins GK, Hibberd PL, Boyer EW (2015). Utilizing an ingestible biosensor to assess real-time medication adherence. J Med Toxicol.

[19] Weinger M, Wiklund M, Gardner-Bonneau D (2010). Handbook of Human Factors in Medical Device Design.

[20] (2016). Applying human factors and usability engineering to medical devices: guidance for industry and food and drug administration staff. U.S. Department of Health and Human Services, Food and Drug Administration.

[21] (2020). ABILIFY MYCITE® (aripiprazole tablets with sensor) - manufactured by Otsuka Pharmaceutical Co. Ltd., Tokyo. US Food and Drug Administration.

[22] Breckenridge A, Aronson JK, Blaschke TF, Hartman D, Peck CC, Vrijens B (2017). Poor medication adherence in clinical trials: consequences and solutions. Nat Rev Drug Discov.

[23] Plowman RS, Peters-Strickland T, Savage GM (2018). Digital medicines: clinical review on the safety of tablets with sensors. Expert Opin Drug Saf.

